# Music speaks louder than lyrics: a conceptual priming experiment

**DOI:** 10.3389/fpsyg.2026.1659797

**Published:** 2026-03-09

**Authors:** Alice Karbanova, Anja-Xiaoxing Cui

**Affiliations:** 1 Department of Romance Languages, Faculty of Arts, Masaryk University, Brno, Czechia; 2 Department of Musicology, Faculty of Philology and Cultural Studies, University of Vienna, Vienna, Austria; 3 Vienna Cognitive Science Hub, University of Vienna, Vienna, Austria

**Keywords:** cognitive linguistics, conceptual priming, musical semantics, music-language interactions, song interpretation

## Abstract

**Introduction:**

Although the processing of language and music are thought to be related, the semantic interplay of these domains in song remains relatively unexplored. This study investigates how music and lyrics contribute to conceptual meaning-making in song interpretation using a conceptual priming experiment.

**Methods:**

Fifty participants completed a lexical decision task in which target words were semantically related either to the music or to the lyrics of an ecologically valid song prime. Reaction times were used to infer semantic alignment.

**Results and Discussion:**

The results showed significantly faster responses to target words associated with the music than to those associated with the lyrics of the prime. This effect remained significant even after controlling for various properties of the primes and targets, which had been assessed by an additional 234 participants in complementary studies prior to the priming experiment. We also found a significant interaction between target type (music- vs. lyrics-related) and the Euclidean distance of valence and arousal between the prime and target: affective distance predicted reaction times only for music-derived targets. Ratings from the complementary studies indicated that music evoked more positive and arousing responses than lyrics, while lyrics appeared to dampen the affective intensity of musical excerpts. Our findings challenge the assumption of tight integration between melody and lyrics in song processing. They suggest that music and language contribute unequally to conceptual interpretation in song, with music playing a more dominant role. These results offer new insights into the construction of multi-modal meanings and the cognitive mechanisms underlying song comprehension.

## 1 Introduction

Although language and music share many structural features and have been extensively studied with respect to shared cognitive processes, research on interactions between their semantic aspects remains relatively scarce. This lack of research is particularly surprising, given the unique fusion of linguistic and musical elements carrying meaning in songs (Peretz et al., 2004), which constitute a musical behavior found in all known human cultures (Mehr et al., 2019). Studies of song perception thus provide a valuable framework for investigating the interrelationship between language, specifically lyrics, and music perception.

Recent research on music-language interactions has been dominated by topics other than semantics (Nayak et al., 2022; Fiveash et al., 2021; Temperley, 2022; Jansen et al., 2023). Existing studies addressing the relationship between music and lyrics specifically have primarily focused on memory and recognition (Crowder et al., 1990; Samson and Zatorre, 1991; Serafine et al., 1984; Peretz et al., 2004), or the relative influence of lyrics and music on mood and perceived emotion (Stratton and Zalanowski, 1994; Ali and Peynircioğlu, 2006; Omigie, 2015). The latter studies can be read as investigating the interplay of semantic processing of lyrics and music, in so far as the expressed emotion is part of the semantic content of both lyrics and music (Steinbeis and Koelsch, 2011).

### 1.1 Processing of emotion in song

An early study examining the relative importance of different emotional sources in music compared singing with instrumental versions (Stratton and Zalanowski, 1994). It found that, in the absence of singing, listeners were unable to identify the intended emotion of the music. Even when the melody was upbeat, the presence of sad lyrics heightened feelings of depression in listeners. The authors concluded that lyrics play a more significant role than vocals or instrumental elements in shaping listeners' emotional responses. These findings align with research suggesting that lyrics also dominate song memory (Peynircioğlu et al., 1998; Serafine et al., 1984).

However, these findings contrast with those of another study that examined the potential dominance of lyrics in shaping affective responses by comparing lyrics without vocals to instrumental music (Sousou, 1997). In this study, participants read happy or sad lyrics while listening to either happy or sad instrumental music. The participants' mood was influenced by background music rather than lyrical content, suggesting that, in the absence of vocal cues, instrumental music exerted a stronger effect on affective responses than lyrics alone. And in yet another study, when a song in an unknown language was presented in which sad lyrics were accompanied by happy music, happiness ratings were greater than sadness ratings. But when the translation of the sad lyrics was also presented on the screen, neither music nor lyrics dominated and ratings of happiness and sadness were statistically indistinguishable (Mori and Iwanaga, 2013).

Similar conclusions were drawn in a separate study (Ali and Peynircioğlu, 2006), which compared the emotional influence of instrumentals with that of vocals and lyrics. Here, the intended emotion of the instrumental track was found to dominate listeners' affective responses, overriding the emotional content of added vocals and lyrics. Notably, the authors also observed an interaction between the emotion conveyed and the presence of vocals and lyrics: when these were emotionally congruent with the instrumental, they intensified sadness but diminished feelings of happiness. Several studies have reported a distinct processing of happy and sad music, whereby the dominance of vocals and lyrics increases in sad music (Brattico et al., 2011; Vidas et al., 2020). However, this distinction is not found in all cultures (Barradas and Sakka, 2022).

### 1.2 Lyrics and melody processing—Integrated or independent?

Research on song perception has also explored whether the concurrently presented auditory streams of lyrics and melody are processed independently or as an integrated whole. Neurological evidence points to both shared and specialized pathways for music and language processing.

The view of integrated processing is buttressed by converging evidence from neuroimaging and behavioral studies. These studies report neural overlap in the processing of speech and music (Rogalsky et al., 2011; Schulze et al., 2011), shared neural circuitry (Tallal and Gaab, 2006), overlapping cortical and subcortical networks (Asaridou and McQueen, 2013), and even neural populations selectively responsive to sung music located adjacent to regions specialized for speech and instrumental music (Norman-Haignere et al., 2022). Additional evidence points to overlapping mechanisms for semantic processing in language and music (Calma-Roddin and Drury, 2020; Miranda and Ullman, 2007), as well as between speech and song (Rossi et al., 2020). Consistent with this view, Samson and Zatorre (1991) found that songs are better remembered when accompanied by lyrics.

Other research however supports the independence hypothesis. Bonnel et al. (2001) found no interference between semantic and melodic processing in a divided attention task, implying that lyrics and melody may be processed as distinct perceptual Gestalts (Bregman, 1990). Likewise, Besson et al. (1998) provided evidence for the independence of linguistic and melodic components in songs. A more nuanced perspective is offered by Sammler et al. (2010), who demonstrated interactive processing of lyrics and melodies in the left middle superior temporal sulcus at a prelexical level, but a functional dissociation in more anterior regions. This supports the idea of a posterior–anterior gradient, along which integration and separation vary. Still, the degree of integration or separation might be task-dependent (LaCroix et al., 2015).

On the whole, the existing literature suggests that the contributions of lyrics and music to emotion processing in song are unequal, thereby supporting the independence hypothesis. As outlined earlier, some older studies indicate that lyrics exert a stronger influence on affective responses (Stratton and Zalanowski, 1994; Peynircioğlu et al., 1998; Serafine et al., 1984), while others find that music plays the leading role (Sousou, 1997; Ali and Peynircioğlu, 2006). More recent studies suggest that the relative influence of lyrics and music may depend on the specific emotional content being conveyed (Brattico et al., 2011; Vidas et al., 2020).

However, these studies face methodological limitations. For instance, research using popular and familiar music (Brattico et al., 2011; Vidas et al., 2020) cannot fully control for genre-specific trends toward positive affect in popular music (Interiano et al., 2018), nor for personal memories associated with familiar songs. Such memories can themselves evoke emotional responses, and when triggered by music, they are often associated with positive affect (Cuddy et al., 2017; Jakubowski et al., 2020; Krumhansl and Zupnick, 2013; Parks and Clancy Dollinger, 2014). Moreover, in some studies, the instrumental stimuli were drawn from instrumental pieces (Ali and Peynircioğlu, 2006; Sousou, 1997), which were never intended to accompany vocals or lyrics. This may have introduced a bias in favor of instrumental dominance in affective responses.

### 1.3 Present investigation

Our study seeks to address these limitations through rigorous stimulus validation, while also extending the focus beyond emotional responses to examine broader conceptual processing in song. While studies investigating event-related potentials suggest that instrumental music can evoke concepts beyond emotion, such as “wideness” (Koelsch et al., 2004) or “battle” (Proverbio et al., 2022), to the best of our knowledge, no study has studied the processing of semantic content when presented simultaneously both in musical and linguistic means.

## 2 Methods

Here, we used a conceptual priming paradigm with relatively unfamiliar and ecologically valid song stimuli composed and written by French polymath Boris Vian. Participants listened to short excerpts from songs and then completed a lexical decision task on a target word that was semantically related either to the lyrical or musical component of the excerpt. We hypothesized that participants would perform faster in the lexical decision task when the target word was conceptually related to the modality which dominates song perception, as conceptual priming should facilitate processing of congruent targets and potentially inhibit incongruent ones. In doing so, we aim to contribute to the ongoing debate on whether music and language processing operate as integrated or independent systems by examining how each modality drives semantic processing of song.

Below, we provide details about the participants, stimuli, procedure, and analysis of the lexical decision task. Within the sections regarding the psychological properties of stimuli, we provide details about additional participants and how the properties were assessed. Given that the language of the presented stimuli was French, only native French speakers were invited to participate in the experiment. All participants gave informed consent. All statistical analyses were performed using RStudio (R Core Team, 2023), and a significance threshold of *p* < 0.05 was used unless stated otherwise. The study procedures were reviewed by the ethics review board of Masaryk University, CZ.

### 2.1 Participants

For the conceptual priming experiment, we recruited 50 participants (31 identified as male; as age was assessed in brackets, we report the median age group of 20–40 years here as well as for the participant groups below). 16 participants completed the experiment in a laboratory setting, while the remaining participants were recruited through mailing lists. For the online group, a brief pre-test meeting was conducted via Zoom to establish rapport and explain the procedure.

Before the lexical decision task, participants completed a demographic questionnaire and an integrated informed consent form on the Gorilla™ platform. Participants also answered a number of *ad hoc* questionnaire items, which aimed to assess subjective bias toward different components of the songs (see Supplementary Table S1). Participants received compensation of 12 euros (or the local currency equivalent) for their participation.

### 2.2 Stimuli—Primes

We selected songs by Boris Vian based on the assumption that, even in France and other French-speaking regions, he is predominantly known as a writer rather than as a musician, and even less so as a composer. We restricted our stimulus set to 13 songs for which Boris Vian was both the lyricist and composer, and for which musical scores were available. The arrangements for voice and piano were obtained from Éditions Jacques Canetti. To the best of our knowledge, several of these songs were recorded for the first time by our team, allowing the musicians a high degree of interpretative freedom during the recording process. Participants' familiarity with Vian's musical work was low: Only 10% of all participants reported being familiar with Vian's music (see Supplementary Table S2). This supports the conclusion that our stimuli were unlikely to evoke personal memories or associations, minimizing potential confounds related to prior exposure.

From the pool of 13 songs, excerpts were selected such that approximately half of the selected excerpts featured passages in which the lyrical and musical semantic content differed. In some cases, different lyrics were set to identical musical accompaniments. These selections were made based on the subjective judgment of AK, and subsequently tested through two preliminary studies (detailed below).

In total, 25 excerpts were selected, each averaging 17 seconds in duration. Each excerpt was recorded in three distinct versions:

1. ML (Music + Lyrics): A complete version combining the sung melody (soprano voice) with piano accompaniment.
2. M (Music only): An instrumental version in which the vocal melody was performed by a viola—a timbre previously shown to evoke voice-like qualities (Proverbio and Piotti, 2022)—with piano accompaniment.
3. L (Lyrics only): A spoken version of the lyrics delivered by a female voice to match the soprano voice from the ML version.

The M and L versions were used to generate target words and were evaluated in preliminary studies regarding a number of variables (detailed below). The ML versions—combining both linguistic and musical information—served as the prime stimuli in the conceptual priming task.

#### 2.2.1 Latent affective properties of recordings

To assess the latent affective dimensions of the musical stimuli, two groups of participants were recruited (54 male, median age group: 20–40 years). One group (*N* = 42) evaluated the ML version while the other group (*N* = 55) evaluated the M and L versions of the excerpts. Because version M contained no linguistic content and version L no musical content, it was highly unlikely that participants would be able to associate excerpts from these two versions with each other. As such, we deemed it methodologically acceptable to use the same group to evaluate both M and L stimuli versions. All participants were recruited online.

Each participant rated the excerpts on a 7-point Likert scale according to two affective dimensions: valence (ranging from unpleasant to pleasant) and arousal (ranging from calm to excited). These dimensions were chosen in accordance with established circumplex models of emotional experience, particularly in the context of music perception, where valence and arousal are known to play a central role (Céspedes-Guevara and Eerola, 2018; Eerola and Vuoskoski, 2011). The goal of collecting these ratings was to assess the potential influence of emotional content on participants' responses in the lexical decision task. Emotional and affective states function as top-down influences that shape perceptual and cognitive processing, guide attention, and facilitate access to emotionally congruent information. In this way, the emotional tone of a stimulus can bias perception and interpretation from the earliest stages of processing.

### 2.3 Stimuli—Targets

To derive appropriate target words for use in the priming task, we recruited two additional groups of participants (*N* = 99, 60 male, median age group: 20–40 years). The subjects were asked to provide free associations in response to auditory excerpts from each of the three stimulus versions. Participants were instructed to list up to four words that spontaneously came to mind while listening to each excerpt. They were encouraged to respond quickly and intuitively, in order to capture immediate conceptual associations and minimize the influence of reflective or strategic thinking. The first group (*N* = 47) evaluated excerpts from version ML, while the second group (*N* = 52) listened to excerpts from versions M and L. All participants were recruited online.

Following data cleaning (lower-casing, lemmatization, and normalization), the most frequently mentioned associations were grouped into semantically homogeneous categories based on their conceptual proximity. This categorization was performed by AK using semantic analysis, which examines the meanings of words and their relationships in order to group them into shared semantic fields, and content analysis, which systematically categorizes words based on emergent thematic criteria to interpret patterns of meaning. Despite potential biases, manual annotation remains a standard and accepted practice in studies of this kind (Bolognesi et al., 2017). From these categorized association fields, the most representative terms were selected as target words for the main experiment (see Supplementary Table S3).

#### 2.3.1 Cosine similarity

To control for the potential influence of semantic congruency between the musical and lyrical components on reaction times in the conceptual priming task, we computed cosine similarity between the sets of associations generated in response to versions M and L. Conceptual similarity can be quantified in various ways, depending on the underlying theoretical framework. In distributional semantic models, for example, word similarity is inferred from linguistic co-occurrence patterns—words that occur in similar contexts tend to share similar meanings (Fernandino et al., 2022). For example, in distributional semantic models, the words doctor and nurse are inferred to be semantically similar because they frequently occur in similar linguistic contexts (e.g., hospital, patient, and treatment), despite differing in form.These models use word vectors that represent lexical items as points in a high-dimensional space, allowing semantic similarity to be measured geometrically.

To implement this analysis, the association data were tokenized and transformed into vector representations using pre-trained embeddings from the open-source FastText library (Joulin et al., 2017), where each word is represented as a 300-dimensional vector based on contextual usage across large text corpora. We performed pairwise cosine similarity comparisons between the association sets generated for the ML and M versions, the ML and L versions, and the M and L versions. The goal was to determine whether the integrated excerpts (ML) semantically aligned more closely with either of their individual components.

Since cosine similarity was calculated using publicly available vectors, we employed repeated-measures ANOVAs. These analyses revealed no significant differences in cosine similarity between any of the version pairings (*F*_(2, 48)_ = 3.32, Greenhouse-Geisser corrected *p* = 0.066). A paired t-test showed that the semantic proximity between targets generated from version ML and version M was statistically indistinguishable from that between version ML and version L (*t*_(22)_ = −0.82, *p* = 0.42, 95% CI [−0.10, 0.043], mean difference = –0.028). This suggests that neither the musical nor the lyrical component alone is a better predictor of the conceptual associations evoked by the combined version.

Overall, the corpus showed a low degree of semantic similarity across associations, indicating that while the concepts were related, they were not highly overlapping. In their study, Hiebel et al. (2022) used a similarity threshold of 0.4, above which concepts are considered semantically close. In our data, only one stimulus in each of the ML–M and L–M comparison exceeded this threshold. For M–L comparisons, only three stimuli reached values above 0.3. Notably, these were also the stimuli we had pre-identified as congruent, validating our initial judgment through quantitative analysis.

#### 2.3.2 Latent semantic properties of the target words

Words have formal and semantic characteristics that may influence the speed and ease with which they can be recognized and understood (Syssau and Font, 2005). It is therefore essential to identify and account for such features when interpreting results from semantic tasks. Fernandino et al. (2022) further demonstrated that conceptual coding theory, which models concepts as grounded in experiential content, outperforms competing models in predicting conceptual behavior. This highlights the importance of incorporating experiential dimensions such as sensory experience and concreteness when investigating how words influence cognitive processes.

To control for the potential influence of such latent semantic properties of target words on participants' responses in the lexical decision task, a new group of participants was recruited online to evaluate the target words on their subjective frequency, valence, arousal, imageability, concreteness, and sensory experience using 7-point Likert scales (*N* = 38, 16 male, median age group: 40–60 years). These variables were selected based on prior research highlighting their relevance in lexical processing and semantic cognition.

To assess how the recording version from which the target word was derived affected semantic ratings, we fitted a linear mixed-effects model for each of the six dimensions, including random intercepts for both the participants and the items. Because mixed-effects models account for inter-individual variability in a statistically robust manner, we did not conduct separate tests of internal consistency or reliability of participants' ratings (Desrochers and Thompson, 2009; Bonin et al., 2003, 2015).

**Figure 1:**
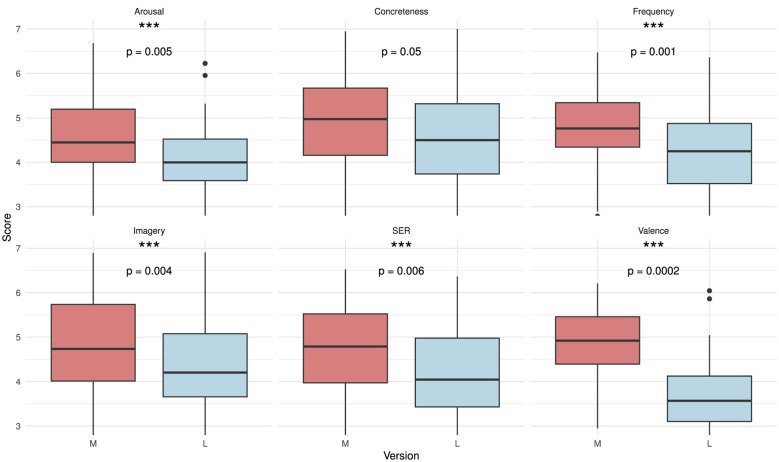
Ratings of music version-derived (M) and lyrics version-derived (L) target words for each latent semantic property. Three asterisks (***) indicate a significant effect.

Setting version M (music only) as the reference level, the results showed a statistically significant decrease in ratings for version L (lyrics only) across all dimensions except concreteness, after Bonferroni correction (α = 0.008). Participants consistently rated targets associated with version L lower than those from version M. These differences are illustrated in Figure 1, with significant effects after alpha correction marked by asterisks. Interestingly, this systematic reduction in ratings for version L is somewhat unexpected, given that the target words themselves do not show any obvious semantic disparities (see Supplementary Table S3).

#### 2.3.3 Lexical properties of target words

Lexical factors such as word length and orthographic neighborhood size are well-established predictors of response times and accuracy in lexical decision tasks (Jiang et al., 2025; Stenneken et al., 2007; Grainger, 1990). Consequently, both experiential and linguistic information must be integrated to form a comprehensive representation of semantic and conceptual knowledge. In addition to semantic variables, we also gathered several objective lexical measures from the Lexique 3 database (New et al., 2004). These included lemma frequency in films, number of letters, number of homographs and homophones, number of syllables, and counts of phonological and orthographic neighbors. The selection of these variables was guided by prior research highlighting their relevance to lexical processing (Hino et al., 2013).

To assess potential differences between target words based on the recording version from which they were derived (M vs. L), we conducted a series of independent-samples *t*-tests. Because these analyses relied on publicly available scores, we could not account for random factors such as participant or item, as was done in other statistical models in this study. After Bonferroni correction for multiple comparisons, none of the differences reached statistical significance.

#### 2.3.4 Pseudo-word targets

In lexical decision tasks, where pseudo-words conform to the orthographic and phonological rules of the target language, participants' decisions rely primarily on the semantic features of real words (Syssau and Laxén, 2012). In contrast, pseudo-words which violate these linguistic conventions are easily dismissed based on superficial features alone, such as letter combinations or pronounceability. Their rejection does not require access to lexical or semantic memory, leading to shallower processing. Following established procedures in the field (e.g., Kousta et al., 2009; Syssau and Laxén, 2012), we constructed orthographically and phonologically legal pseudo-words by altering a single vowel in each target word (see Supplementary Table S3). This method ensured that the resulting pseudo-words were pronounceable, orthographically legal and matched in length (number of syllables) with the real experimental prime.

### 2.4 Procedure

We employed a cross-random-effects design using a lexical decision task, in which participants judged whether a given letter string was a real word or a pseudo-word. The primary objective was to compare reaction times (RTs) to real target words originating from excerpt versions M and L, presented in response to the ML recordings which were the primes. RTs were interpreted as an index of conceptual proximity between prime and target—shorter RTs were taken to reflect a stronger semantic association.

We randomized the trial order for each participant. During each trial, a fixation cross appeared at the center of the screen where the target word would subsequently appear. Participants were instructed to fixate on the cross to minimize eye movements and enable faster responses. They were asked to decide as quickly as possible whether the displayed word was a real French word (pressing the “right” arrow key) or a pseudo-word (pressing the “left” arrow key). If a response took longer than 500 ms, a “Answer faster!” prompt appeared.

Following each trial, a burst of white noise was played. This served two purposes: to mask the auditory trace of the preceding excerpt and to function as a pacing mechanism. Participants could only proceed after pressing the space bar, ensuring attentiveness and allowing them to advance at their own pace. They were instructed to keep their right-hand fingers positioned on the “left” and “right” arrow keys throughout the task to enable rapid responses.

The estimated duration of the test was approximately 45 min. However, since participants progressed at their own pace, the actual durations varied. We provided headphones for in-lab participants and encouraged remote participants to use headphones as well.

### 2.5 Analysis

**Figure 2:**
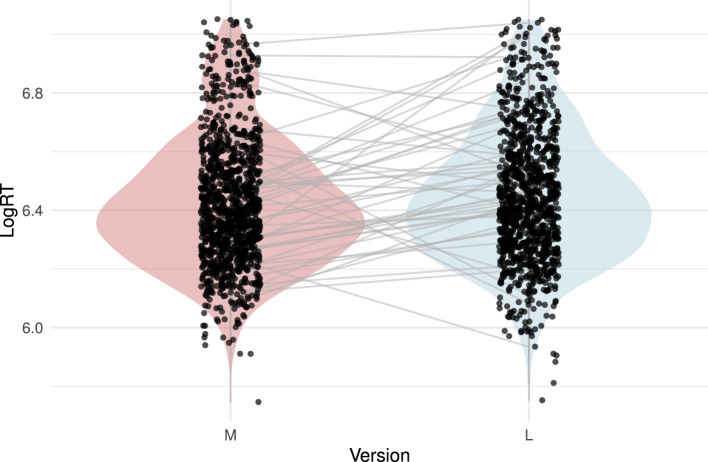
Logarithmically transformed reaction times to music version-derived (M) and lyrics version-derived (L) target words (outliers removed).

Our first analysis examined the central question of whether musical or lyrical meanings are accessed more quickly when participants are primed with a song. To assess this, we first log-transformed the RTs in the lexical decision task to reduce positive skew and approximate a normal distribution, in accordance with standard practice in the field (Kousta et al., 2009). Prior to statistical analysis, we removed outliers using the IQR method. Figure 2 presents violin plots summarizing the distribution of log-transformed RTs.

Next, we accounted for a possible influence of the prime properties by controlling for their inner congruency using two separate scores: the Euclidean distance and the cosine similarity between their two constituting components, that is, the distance between versions M and L as recordings in the Euclidean distance score, and as conceptual associations in the cosine similarity score. We further controlled for a possible influence of target word properties, namely, the latent semantic properties (Section 2.3.2) and the lexical properties (Section 2.3.3). We also controlled for a possible influence of the semantic distance between prime and target and a potential interaction between these two effects. In order to account for individual differences between participants, we first explored the correlation between their preferences toward one or the other song component in song listening, as self-assessed in an *ad hoc* questionnaire, and their implicit bias, as indexed using their RTs. Second, we evaluated the possible influence of individual variability of participants by controlling for the effect of demographic variables.

## 3 Results

### 3.1 Effect of stimulus version on affective ratings

**Figure 3:**
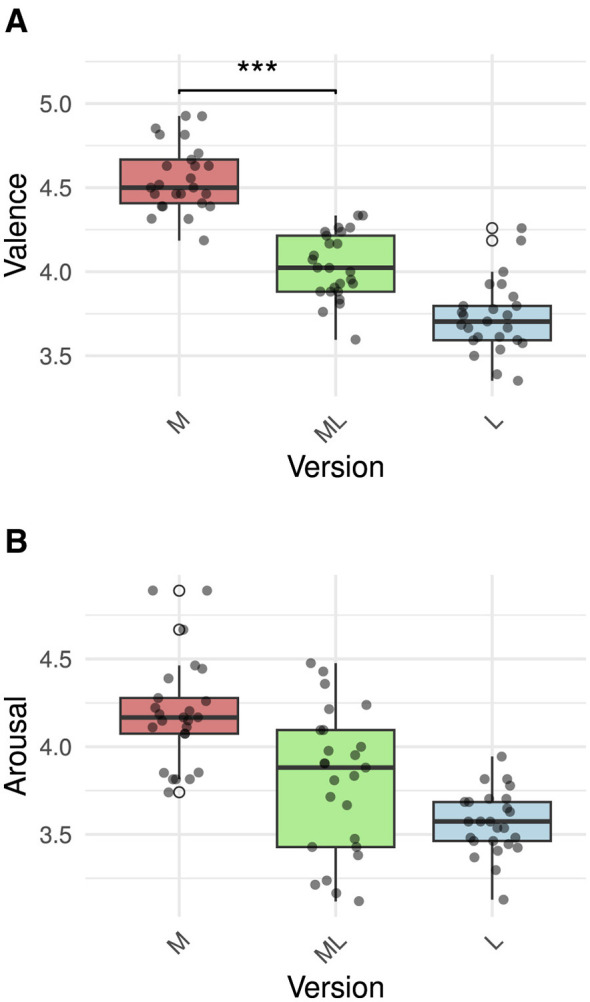
Latent affective properties of the three recording versions. Three asterisks (***) indicate a significant effect. **(A)** Valence ratings. **(B)** Arousal ratings.

To assess the effect of stimulus version on affective ratings, we fitted a maximum likelihood mixed-effects model with random intercepts for both participants and items (see Figure 3). The reference condition was set to version ML. For valence ratings, version M was associated with significantly higher ratings compared to version ML (β = 0.54, *p* = 0.023), Bonferroni-corrected α = 0.025 while version L showed a weaker and non-significant effect (β = −0.32, *p* = 0.175). For arousal ratings, while the trend remained the same, neither of the comparisons was significant (version M: β = 0.44, *p* = 0.089, version L: β = −0.23, *p* = 0.3).

### 3.2 Effect of target version

We fitted a linear mixed-effects model using maximum likelihood estimation to examine the effect of target version on RTs, while accounting for random intercepts for participants and primes. A random slope for participants was not included due to convergence issues and model overfitting.

The model demonstrated good fit (AIC = −1, 476.9, BIC = −1, 449.0, log-likelihood = 743.5). Random effects showed a variance of 0.034 for subject intercepts and 0.002 for the primes, while the residual variance was 0.025. The fixed-effect intercept was estimated at 6.456, while the effect of the version L-derived target words (lyrics associated concept) was estimated at 0.0294 (*p* < 0.001), reflecting a statistically significant increase in RTs relative to version M-derived target words (music associated concept), corresponding to 17 ms (see Table 1).

**Table 1:** Summary table of the influence of the latent semantic and lexical properties of target words on the effect of version.

**Model**	Reaction time (ms)			**95% CI (ms)**	**SE (ms)**	**df**	**t**	**p**
	**M**	**L**	Δ					
Basic	637	655	+17	646–663	7.1	1989	3.91	< 0.001^*^
+ Arousal	646	664	+18	653–675	8.7	1523	3.27	0.001^*^
+ Concreteness	615	635	+20	625–645	8.2	1558	3.99	< 0.001^*^
+ Subjective Frequency	687	701	+14	687–714	9.9	418	2.06	0.040
+ Imageability	669	686	+17	674–698	8.9	1247	2.88	0.004
+ Sensory Experience	653	671	+18	660–682	8.9	1167	3.06	0.002^*^
+ Valence	645	662	+17	650–674	9.6	1478	2.78	0.006
+ Film Frequency	722	741	+19	731–752	0.01	1920	3.70	< 0.001^*^
+ Letters	602	619	+16	610–628	0.01	1929	3.69	< 0.001^*^
+ Homographs	651	670	+19	661–680	0.01	1929	4.10	< 0.001^*^
+ Homophones	644	664	+20	655–674	0.01	1929	4.29	< 0.001^*^
+ Orth. Neighbors	640	659	+19	650–669	0.01	1929	4.09	< 0.001^*^
+ Phon. Neighbors	641	660	+19	650–669	0.01	1929	3.99	< 0.001^*^
+ Syllables	572	590	+18	582–599	0.01	1924	4.43	< 0.001^*^

The estimates show LogRTs transformed back into milliseconds for better interpretation. Significant effects after Bonferroni correction (*p* < 0.004) are marked with an asterisk (^*^). The *p* value column indicates whether the effect of version remains significant after controlling for different other variables.

To validate the contribution of the target version predictor, we also fitted a reduced model without this variable. A likelihood ratio test between the two models confirmed a significant difference ($\chi_{\left(1\right)}^{2}=16.756,\text{p}<0.001$), underscoring the explanatory power of target version.

The marginal $R_{m}^{2}=0.0036$ and conditional $R_{c}^{2}=0.596$, calculated using the r.squaredGLMM function from the MuMIn package (Bartoń, 2024), indicated that while the fixed effect accounted for a small portion of the variance, the random effects contributed substantially. Cohen's *d*, calculated manually, was 0.57, indicating a moderate effect size.

We estimated confidence intervals using the confint function from the lme4 package (Bates et al., 2015). The 95% CI for the intercept (version M-derived target words) ranged from 6.40 to 6.51, reflecting a precise estimate. For version L-derived target words, the 95% CI ranged from 0.015 to 0.043, suggesting a small but reliable positive effect, as the interval does not include zero.

### 3.3 Effect of inner congruency of the primes

To assess the possible influence of prime properties on the semantic recognition of targets, we calculated its inner semantic congruency score based on each excerpt's position in a two-dimensional space defined by valence and arousal ratings (see Section 2.2.1). The effect of inner congruency was not statistically significant (*p* = 0.156). Similarly, we controlled for the congruency between M and L versions of the prime excerpts using the cosine similarity scores (Section 2.3.1). Adding this predictor did not yield a significant effect either (*p* = 0.867).

We further tested a congruency hypothesis from earlier studies, including Ali and Peynircioğlu (2006), which reported that emotionally congruent combinations of lyrics and melody intensified perceived emotion. In our study, we used Euclidean distance in valence–arousal space as a proxy for congruence between excerpt version M and L and examined its effect on excerpt version ML ratings. While Euclidean distance did not predict valence ratings (*p* = 0.839; *R*^2^*m* = 0.0018), it significantly predicted arousal ratings (*p* = 0.0304; *R*^2^*m* = 0.188), indicating that greater incongruence between music and lyrics was associated with increased emotional arousal.

### 3.4 Effect of latent semantic properties of the target words on RTs

To account for the semantic characteristics of the target words, we initially attempted to fit a full mixed-effects model including all latent semantic properties as fixed effects, along with target version and random intercepts for participants and primes. However, due to multicollinearity among the semantic variables, we instead ran separate models for each one, using version M-derived target words as the reference level.

Across all models, the estimated effect of target version changed slightly but remained statistically significant in all but the models controlling for subjective frequency, imagebility and valence (see Table 1 for the estimates for the effect of version in the basic model and the models with additional predictors). To evaluate the impact of these three predictors, we conducted ANOVA model comparisons showing that none of those models significantly improved model fit (see Table 2).

**Table 2:** Model comparisons (likelihood ratio tests) assessing the effect of additional predictors beyond Version.

**Model**	**AIC**	**BIC**	**logLik**	**χ^2^**	**df**	**p**
Basic	–1,477.0	–1,449.0	734.47			
+ Subjective frequency	–1,476.6	–1,443.0	744.29	1.63	1	0.202
+ Imageability	–1,475.7	–1,442.1	743.85	0.75	1	0.388
+ Valence	–1,475.2	–1,441.7	743.62	0.29	1	0.588
+ Film frequency	–1,490.5	–1,457.0	751.26	15.57	1	< 0.001^*^
+ Syllables	–1,490.3	–1,456.8	751.15	15.35	1	< 0.001^*^

Significant effects after Bonferroni correction (*p* < 0.01) are marked with an asterisk (^*^). The *p* value column indicates whether the model with the added predictor was significantly improved over the basic model.

Overall, none of the latent semantic predictors accounted for additional variance in RTs beyond that explained by target version. The RT difference between versions M and L thus remained robust even when controlling for these semantic factors.

### 3.5 Effect of lexical properties of target words on RTs

To evaluate the impact of the lexical properties of the target words on reaction times, we fitted mixed-effects models with random intercepts for participants and primes. The target version effect remained statistically significant in all models (see Table 1).

Only two models yielded additional significant effects, namely lemma frequency in films and number of syllables. Firstly, the lemma frequency in films had a significant negative effect on RTs (*t*_(73)_ = −4.515, *p* < 0.001), indicating faster responses to more frequent words (*R*^2^*m* = 0.014, *R*^2^*c* = 0.59). Model comparisons confirmed that including the frequency in films predictor significantly improved model fit (see Table 2). Type III ANOVA indicated that both frequency in films and version predictors contributed significantly, with the frequency in films exerted a slightly greater impact (*F* = 20.39 for the frequency in films vs. *F* = 13.39 for target version), consistent with prior psycholinguistic findings on word frequency.

Secondly, the number of syllables had a significant positive effect on RTs (*t*_(29)_ = 4.383, *p* < 0.001), indicating slower responses to words with more syllables. Type III ANOVA revealed that number of syllables accounted for variance comparable to the target version effect (*F* = 19.20 vs. 19.60). Model comparisons confirmed improved fit with this predictor (see Table 2).

### 3.6 Effect of semantic distance between primes and target words

In order to control for a possible influence of the semantic distance between primes, that is, the ML recordings, and target words, that is, concepts derived from M or L recordings, we calculated their Euclidean distance using the valence and arousal scores obtained from pre-testing (for primes see Section 2.2.1, for target words see Section 2.3.2). A paired t-test showed that the difference between the two distances, that is, ML prime to version L-derived target word vs. ML prime to version M-derived target word, was not statistically significant (*t*_24_ = 1.82 *p* = 0.081).

Adding the semantic distance predictor to the model revealed its significant influence on the RTs (*t*_(523)_ = 2.058, *p* = 0.04). The further apart the affective profile of the target word is from the prime, the longer it takes participants to respond, suggesting that emotional distance between the prime and the target makes processing less efficient. The type III ANOVA showed that the effect of version remained statistically significant even when controlling for the semantic distance (*F*_(1, 1, 999.81)_ = 19.16, *p* < 0.001). Given that target words derived from version M recordings tended to be significantly higher rated on valence and arousal than target words derived from version L recordings (see Section 2.3.2), we also checked for a potential interaction between the effect of target version and semantic distance. The interaction was significant as confirmed by type III ANOVA (*F*_(1, 1, 679.89)_ = 6.13, *p* = 0.013) suggesting that the effect of semantic distance on RTs differs between version M- and version L-derived target words.

**Figure 4:**
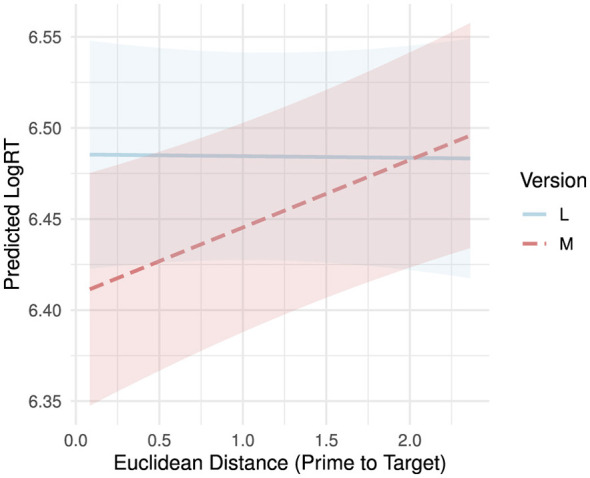
Result of the simple slopes analysis of the interaction between the effect of version and Euclidean distance. Model-based 95% confidence intervals show the uncertainty around predicted LogRTs across Distance values, separately by Version, reflecting the expected range for the true mean given the model and data.

To follow up the significant interaction between target version and semantic distance on RTs, we conducted a simple slopes analysis using estimated marginal trends from the interactions package (Long, 2024). The analysis revealed that semantic distance had a significant positive effect on RTs in version M (b = 0.037, 95% CI [0.0135, 0.0606]) indicating that participants responded faster to the version M-derived target words when they were affectively closer to the primes (ML recordings). In contrast, no such effect was observed for version L-derived target words (b = −0.0009, 95% CI [−0.027, 0.025]) suggesting that emotional distance from the primes did not influence reaction times when the target word was derived from a lyrics only recording (see Figure 4).

### 3.7 Implicit and explicit bias in song perception

Participants' explicit biases were measured using responses from the self-report questionnaire (see Supplementary Table S1). Preference scores were mean-centered, such that positive values indicated a bias toward M and negative values toward L. To assess implicit bias, we computed the difference in participants' log-transformed RTs between version M- and version L-derived target words. This RT difference served as an index of automatic, implicit preference for one component over the other.

**Figure 5:**
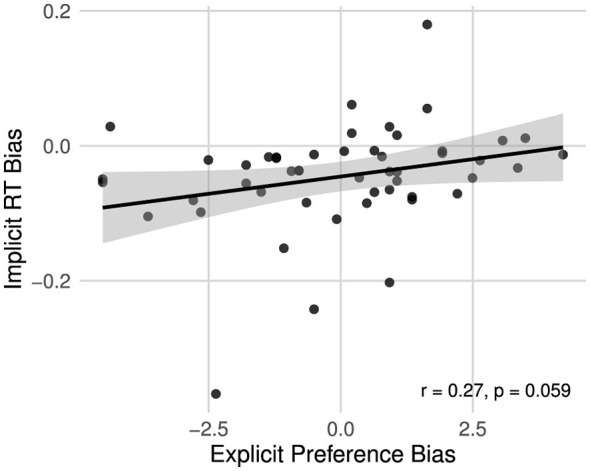
Correlation between explicit and implicit bias measures.

A Pearson correlation between explicit and implicit bias measures revealed a weak but positive association (*r* = 0.269, *p* = 0.059), suggesting that participants who explicitly preferred lyrics also tended to show slower responses to version L-derived target words, although the relationship was modest (see Figure 5).

### 3.8 Effect of demographic variables

Finally, we explored whether demographic variables (sex, age, education level, musical experience, and music liking) could explain additional variance by incorporating them as predictors. None of these variables showed a statistically significant effect, indicating that individual differences of participants did not account for more variance than the main condition effect.

## 4 Discussion

While previous research has suggested a tight integration of melody and lyrics in priming paradigms (Gordon et al., 2010), our findings indicate that musical components exert a stronger influence on conceptual processing than lyrical ones. By examining how each modality contributes to semantic priming, we add to the ongoing discussion of whether music and language engage integrated or independent cognitive systems. The observed processing advantage for music suggests that listeners access the semantic content of musical information more quickly than that of lyrics. This effect persisted even after controlling for a wide range of lexical and semantic characteristics of the target words, indicating that it cannot be explained by surface-level word properties alone (Syssau and Font, 2005; Stenneken et al., 2007). These results highlight music's capacity to evoke conceptual meaning independently and efficiently, and point to a potential asymmetry in how music and language contribute to the semantic interpretation of song.

Our findings align with studies that highlight the conceptual and affective richness of music (Ali and Peynircioğlu, 2006; Küssner and Eerola, 2019; Margulis et al., 2022; Koelsch, 2011) and provide further evidence that music is not a mere carrier of affect but can independently generate meaningful semantic associations. While earlier studies have merely debated whether music or lyrics dominate affective responses in song (Stratton and Zalanowski, 1994; Sousou, 1997; Brattico et al., 2011), our results demonstrate a clear behavioral advantage for music-based conceptual processing in song interpretation.

While earlier studies have often focused on structural or perceptual aspects of the music–language interface, our results suggest that a degree of independence also extends to the semantic level. However, affective scores of the recordings collected in a pre-test session point to partial convergence: music and lyrics together produced emotional responses that were more moderate than either component alone (see Section 2.2.1). Strikingly, the presence of lyrics consistently reduced both the intensity and positivity of emotional responses. Overall, version M tended to receive higher ratings in both valence and arousal, while version L was rated lower on these dimensions compared to the stimulus that combined both music and lyrics. These findings support previous results by Ilie and Thompson (2006), who reported that purely musical stimuli elicited more positive and more intense emotional responses than vocal or spoken stimuli. Our results suggest that the presence of lyrics may dampen both the emotional intensity and the perceived positivity of musical excerpts. This aligns with prior work suggesting that lyrics can increase perceived complexity and dampen enjoyment (Gfeller and Coffman, 1991), and that melodies alone evoke stronger emotional responses than when paired with lyrics (Ali and Peynircioğlu, 2006). One possibility is that lyrics add cognitive and emotional complexity, or reduce emotional clarity especially if comprehension is hindered. Indeed, Johnson et al. (2013) found that sung lyrics, particularly melismatic ones, impair verbal recognition compared to spoken text. That lyrics may weaken music's emotional impact is counterintuitive and merits further exploration.

Importantly, our study addressed limitations in prior research by using unfamiliar song material, minimizing the influence of personal memories and genre expectations (Cuddy et al., 2017; Jakubowski et al., 2020; Krumhansl and Zupnick, 2013; Parks and Clancy Dollinger, 2014). We assessed participants' familiarity with the musical work of Boris Vian using a demographic questionnaire, which showed that only 10% of participants were familiar with it. Critically, to the best of our knowledge, some of the songs used in this study were recorded for the first time by our team, meaning that prior exposure is unlikely. At the same time, the focus on the French language represents one of the strengths of this study, as it helps counterbalance the over-representation of English in the cognitive sciences (Blasi et al., 2022). On the other hand, despite efforts to enhance ecological validity by using real music excerpts, the experimental setting and the laboratory presentation of song snippets differ substantially from real-life listening experiences, and the results should therefore be interpreted with caution.

The collection of affective scores of each type of stimuli and carefully controlled lexical and semantic target properties strengthen the internal validity of our findings. Additionally, our methodological innovations, such as computing inner congruency of song excerpts using Euclidean distance in affective space and cosine similarity in semantic space, provide a novel approach to quantifying semantic alignment in multimodal stimuli. Crucially, we explored broader conceptual processing and thereby investigated semantic aspects beyond emotion processing in song (Mori and Iwanaga, 2013; Vidas et al., 2020; Brattico et al., 2011). While previous studies have indicated that instrumental music can evoke semantic concepts beyond emotion (Proverbio et al., 2022; Koelsch et al., 2004), here we show that such concepts may be processed preferentially over concepts evoked by lyrics at the same time.

Building on prior work (e.g., Ali and Peynircioğlu, 2006), we further tested a congruency hypothesis by operationalizing music–lyrics congruence as Euclidean distance in valence–arousal space between excerpt versions M and L. We examined whether this distance predicted ML ratings and found that congruence did not explain valence judgments, whereas greater music–lyrics incongruence was associated with heightened emotional arousal. This contradicts Ali and Peynircioğlu (2006)'s findings and suggests that emotional dissonance may amplify arousal, rather than diminish it. However, these findings are consistent with those of Stratton and Zalanowski (1994), who found that combining pleasant music with negative lyrics can intensify emotional impact through a cognitive dissonance mechanism, requiring listeners to reconcile conflicting cues. This effort to resolve incongruity may enhance emotional engagement.

Furthermore, we found that the semantic distance between the affective profiles of the prime and the target—expressed as Euclidean distance—significantly predicted reaction times, but only for version M-derived targets. Greater affective distance resulted in longer response times, indicating that affective congruence facilitates lexical access when the target word was conceptually related to the musical aspect of the prime. The absence of this effect when the target word was conceptually related to the lyrics of the prime suggests that lyrics may not be processed with the same level of affective integration, or that their affective content is less salient when stripped of musical context. This dissociation provides new insight into how emotional and conceptual processing may differ between musical and linguistic modalities within song (Ali and Peynircioğlu, 2006). Music-derived targets appear more sensitive to affective mismatch with the primes, further supporting the idea that music conveys rich affective meaning that interacts dynamically with subsequent cognitive processing (Boltz, 2001). This is in line with embodied and grounded cognition theories, which propose that affective states shape conceptual processing (Fernandino et al., 2022). This again provides support to studies that have found music and lyrics processing independent (Besson et al., 1998).

We also found that participants' explicit preferences for music or lyrics, as assessed through a *ad hoc* questionnaire items, showed a weak positive correlation with their implicit processing bias in reaction times. While not statistically significant, this trend hints at an individual difference factor that could be explored in future research, especially in studies that aim to personalize or tailor music-based interventions.

In sum, our findings challenge the assumption that lyrics dominate song interpretation (Stratton and Zalanowski, 1994; Peynircioğlu et al., 1998; Serafine et al., 1984) and reveal the complex, sometimes unexpected interplay between music and language. Future work should further explore the mechanisms of emotional blending and processing interference between modalities, especially under naturalistic listening conditions. Taken together, these results provide compelling evidence that musical information can exert a stronger and more immediate effect on conceptual processing than lyrics. While not dismissing the role of language in song, our findings indicate that music and language engage overlapping but distinct interpretative pathways, and that their integration in song may be more asymmetrical than previously assumed.

Lastly, it should be noted that although we chose the stimulus material to eliminate what we perceived to be limitations in previous studies, the selected stimuli present their own limitations regarding the generalizability of our findings: they are from a particular genre, in a particular language, composed and written by a particular person, and perceived by particular listeners. Future research should therefore examine how familiarity, genre and individual listener characteristics modulate the balance between music and lyrics in semantic processing.

## 5 Conclusion

This study provides compelling evidence that music can independently and efficiently drive conceptual processing in song, often more so than lyrics. By demonstrating a consistent reaction time advantage for music-based targets—even when controlling for semantic and lexical factors—we highlight a meaningful asymmetry in how musical and linguistic components contribute to song interpretation. Our findings challenge assumptions of lyrical dominance and support the view that music and language, while often integrated in song, engage distinct interpretive processes. These results underscore music's capacity to convey rich semantic and affective content, advancing our understanding of song perception and open new avenues for exploring the interplay of affect, semantics, and modality in human cognition and offering a foundation for future research into the complex dynamics of multimodal communication.

## Data Availability

The raw data supporting the conclusions of this article will be made available by the authors, without undue reservation.
