# Supplementary material for: Music speaks louder than lyrics: a conceptual priming experiment
Source: Front Psychol. 2026 Mar 9;17:1659797. doi: 10.3389/fpsyg.2026.1659797 (PMC13006578; doi:10.3389/fpsyg.2026.1659797)
Supplement: Supplementary file 1 [file Data_Sheet_1.pdf]

Supplementary Material

1 SUPPLEMENTARY TABLES

Table S1. Explicit bias in song listening: Question ID in the order of presentation, responses and their English translations.

ID	Response (French)	Response (English)
ML3	Si une chanson a une bonne mélodie, les paroles m'importent peu.	If a song has a good melody, I don't care much about the lyrics.
ML28	La mélodie est la partie la plus importante d'une chanson.	The melody is the most important part of a song.
ML35	Quand j'écoute une nouvelle chanson, je porte une attention particulière à la mélodie.	When I listen to a new song, I pay particular attention to the melody.
ML5	Les paroles sont la partie la plus importante d'une chanson.	Lyrics are the most important part of a song.
ML13	Si j'écrivais une chanson, je commencerais par les paroles.	If I were writing a song, I would start with the lyrics.
ML17	Quand j'aime une chanson, c'est souvent à cause des paroles.	When I like a song, it's often because of the lyrics.
ML26	Selon le genre de musique, je porte une attention particulière à la mélodie.	Depending on the genre of music, I pay particular attention to the melody.
ML14	Quand j'aime une chanson, c'est souvent à cause de la mélodie.	When I like a song, it's often because of the melody.
ML15	Si j'écrivais une chanson, je commencerais par la mélodie.	If I were writing a song, I would start with the melody.
ML34	Cela ne me dérange pas si je ne peux pas comprendre correctement les paroles d'une chanson. Je l'aime toujours autant bien.	I don't mind if I can't understand the lyrics of a song correctly. I still like it just as much.
ML25	Si une chanson a de bonnes paroles, la mélodie m'importe peu.	If a song has good lyrics, I don't care much about the melody.
ML27	Selon le genre de musique, je porte une attention particulière aux paroles.	Depending on the genre of music, I pay particular attention to the lyrics.
ML39	Quand j'écoute une nouvelle chanson, je porte une attention particulière aux paroles.	When I listen to a new song, I pay particular attention to the lyrics.
ML47	Je partage souvent des chansons avec mes amis parce que j'aime les paroles.	I often share songs with my friends because I like the lyrics.

Table S2. Familiarity question ”Connaissez-vous l’œuvre musicale de Boris Vian ?” (”Do you know Boris Vian’s musical work?”) and responses with English translation. The number of participants and their level of familiarity with the musical work of Boris Vian per response option number.

Response Option		No. of Participants per Study		
**Original**	**Translation**	**Priming**	**Concepts**	**Affective Norms**
1) Oui, je connais bien ses chansons	Yes, I know his songs well	4	15	6
2) Un peu, une ou deux chansons	A little, one or two songs	7	21	20
3) J’en ai une vague idée, je sais qu’il n’était pas uniquement écrivain mais aussi musicien	I have a vague idea, I know he was not only a writer but also a musician	16	28	37
4) Pas du tout	Not at all	23	35	34

Table S3. Target words with their English translations and corresponding pseudowords. A total of 25 primes were derived from 13 different songs, so some stimuli originate from the same song and therefore share the same number.

Prime	M	M English	L	L English	pseudoword
1	calme	calm	crime	crime	clime
2a	poursuite	pursuit	croyance	belief	misare
2b	printemps	spring	misère	misery	dopart
3a	aventure	adventure	résistance	resistance	abandan
3b	aventure	adventure	trahison	treachery	rosistance
3c	printemps	spring	vengeance	vengeance	parsuite
4a	retrouvailles	reunion	abandon	abandon	salitude
4b	espoir	hope	départ	departure	rapos
5	repos	rest	couple	couple	prontemps
6a	solitude	solitude	confession	confession	oventure
6b	paysage	landscape	abandon	abandon	rapression
6c	printemps	spring	fierté	pride	bogarre
7a	sérénité	serenity	prostitution	prostitution	sarénité
7b	apéro	drink	répression	suppression	abandan
8	festin	feast	bagarre	fight	fiarté
9a	légende	legend	voyou	rascal	imposse
9b	sourire	smile	violence	violence	cametière
10a	regret	regret	impasse	deadlock	sourore
10b	cimetière	cemetery	prostituée	prostitute	lagende
11a	soirée	party	contrôle	control	haver
11b	rencontre	encounter	violence	violence	campogne
12a	prière	prayer	vieillesse	old age	rogret
12b	prière	prayer	hiver	winter	contrale
13a	regret	regret	romance	romance	priore
13b	hiver	winter	campagne	countryside	rincontre
